# The Effect of Interleukin-4 and Dexamethasone on RNA-Seq-Based Transcriptomic Profiling of Human Podocytes: A Potential Role in Minimal Change Nephrotic Syndrome

**DOI:** 10.3390/jcm10030496

**Published:** 2021-02-01

**Authors:** Jiwon M. Lee, Younhee Ko, Chul Ho Lee, Nara Jeon, Keum Hwa Lee, Jun Oh, Andreas Kronbichler, Moin A. Saleem, Beom Jin Lim, Jae Il Shin

**Affiliations:** 1 Department of Pediatrics, Chungnam National University Hospital and College of Medicine, Daejeon 35015, Korea; jwmleemd@gmail.com; 2 Division of Biomedical Engineering, Hankuk University of Foreign Studies, Gyeonggi-do 17035, Korea; younko@hufs.ac.kr; 3 Department of Pediatrics, Yonsei University College of Medicine, Seoul 03722, Korea; CHEOLHOLEE@yuhs.ac (C.H.L.); AZSAGM@yuhs.ac (K.H.L.); 4 Division of Clinical Genetics, Severance Children’s Hospital, Seoul 03722, Korea; 5 Department of Pathology, Yonsei University College of Medicine, Seoul 03722, Korea; REESE722@yuhs.ac; 6 Department of Pediatrics, University Hamburg-Eppendorf, 20246 Hamburg, Germany; j.oh@uke.de; 7 Department of Internal Medicine IV (Nephrology and Hypertension), Medical University Innsbruck, 6020 Innsbruck, Austria; Andreas.Kronbichler@i-med.ac.at; 8 Children’s and Renal Unit and Bristol Renal, University of Bristol, Bristol BS2 8BJ, UK; m.saleem@bristol.ac.uk; 9 Division of Pediatric Nephrology, Severance Children’s Hospital, Seoul 03722, Korea; 10 Institute of Kidney Disease Research, Yonsei University College of Medicine, Seoul 03722, Korea

**Keywords:** nephrotic syndrome, transcriptome, RNA-sequencing, interleukin, gene, podocytes

## Abstract

Interleukin-4 (IL-4) expression is implicated in the pathogenesis of nephrotic syndrome (NS). This study aimed to investigate the changes in the transcriptomes of human podocytes induced by IL-4 treatment and to analyze whether these changes could be affected by simultaneous steroid treatment. Three groups of human podocytes were treated with control, IL-4, and IL-4 plus dexamethasone (DEX), respectively. We performed whole-transcriptome sequencing to identify differentially expressed genes (DEGs) between the groups. We investigated relevant biological pathways using Gene Ontology (GO) enrichment analyses. We also attempted to compare and validate the DEGs with the genes listed in PodNet, a literature-based database on mouse podocyte genes. A total of 176 genes were differentially expressed among the three groups. GO analyses showed that pathways related to cytoskeleton organization and cell signaling were significantly enriched. Among them, 24 genes were listed in PodNet, and 12 of them were previously reported to be associated with IL-4-induced changes in human podocytes. Of the 12 genes, the expression levels of *BMP4*, *RARB*, and *PLCE1* were reversed when podocytes were simultaneously treated with DEX. In conclusion, this study explored changes in the transcriptome profiles of human podocytes treated with IL-4. Few genes were reported in previous studies and were previously validated in experiments with human podocytes. We speculate that IL-4 may exert pathogenic effects on the transcriptome of human podocytes, and a few genes may be involved in the pathogenesis.

## 1. Introduction

Minimal change nephrotic syndrome (MCNS) is the major form of nephrotic syndrome (NS) reported in children, which includes steroid-sensitive NS (SSNS) [1,2,3]. As most primary MCNS cases exhibit a high response rate to corticosteroids, its pathogenesis has been speculated to be immune-mediated, especially involving T cell-associated mechanisms [1,4]. Therefore, multiple studies have been conducted to explore the immunological basis of NS [5,6,7,8,9,10,11,12,13], and several studies specifically suggested T-helper 2 lymphocyte (Th2)-dependency by demonstrating over-activity of type 2 helper T cells (Th2) and decreased Th1/Th2 ratios, thus highlighting Th2 predominance in NS [8,14,15]. Therefore, more studies have been performed with an aim to validate the role of Th2 cytokines such as interleukin (IL)-4, IL-5, and IL-13 in NS [16,17,18,19,20,21,22,23,24].

Among these cytokines, the expression of only IL-4 and IL-13 has mostly been explored because IL-5 expression is reportedly eosinophil-specific. Yap et al. examined the mRNA expression profiles of IL-2, interferon-γ, and IL-13 in 55 children with steroid-sensitive NS and reported increased cytoplasmic IL-13 expression in patients during relapse compared to remission [23]. Further, van den Berg et al. examined the role of IL-4 and IL-13 in human and rat glomerular visceral epithelial cells (GVECs) and showed that IL-4 and IL-13 could exert direct effects on podocytes by binding to specific IL-4 and IL-13 receptors. They also decreased transepithelial electrical resistance of monolayers of rat GVEC to approximately 30% and 40% of baseline values, respectively, in a dose-dependent manner [20].

Moreover, the increased activity of IL-4 has been reported in correlation with the activity of NS [14,21]. Youssef et al. investigated cytokine profiles in 52 children with steroid-sensitive NS and found that serum levels of IL-4, along with immunoglobulin (Ig) E, IL-13, and tumor necrosis factor (TNF)-α were significantly increased during the active phase of proteinuria compared to the remission phase [14]. Prasad et al. studied the T-cell phenotypes and their respective cytokine profiles in children with NS and discovered that the levels of suppressive cytokines, such as IL-10 and transforming growth factor (TGF)-β, decreased in the stimulated peripheral blood lymphocytes at relapse of NS and that these levels increased during remission [25]. Conversely, the expression of stimulatory cytokines, such as IL-4 and interferon (IFN)-γ, increased during relapse and decreased during remission [25]. This study also suggested the role of regulatory T cells (Tregs) by demonstrating that the frequency of Tregs decreased at relapse and increased at remission [25]. Youn et al. also reported increased serum levels of IL-4 and IL-5 in 32 children with NS, which decreased to normal levels after steroid therapy [26], suggesting their pathogenic role in the development of NS. Moreover, a recent in vivo study showed that the overexpression of IL-4 in mice was sufficient to induce kidney injury and result in proteinuria [17]. In this study, the transfer of antigen-specific B cells induced glomerular injury and caused proteinuria, but the transfer of IL-4-deficient B cells did not lead to the development of proteinuria, demonstrating an important role of IL-4 in the pathogenesis of NS. This study also presented evidence that IL-4 induced podocyte membrane ruffling and widespread foot process retraction in murine podocytes [17].

Based on the accumulated evidence on the pathogenic role of IL-4 in NS, we speculated that IL-4 might induce changes in the protein expression in human podocytes. First, we examined the effect of IL-4 on intracytoplasmic actin filaments by immunofluorescence microscopy. We then performed transcriptome analysis in human podocytes and investigated the changes in gene expression levels induced by IL-4. We also examined whether these changes could be reversed by simultaneous steroid treatment. To our knowledge, this is the first study to explore the effect of IL-4 on human podocytes by transcriptome profiling.

## 2. Materials and Methods

### 2.1. Cell Culture of Human Podocytes

Conditionally immortalized human podocytes (AB8/23), primarily cloned from human glomerular cultures, were characterized and generously provided by Dr. Moin A. Saleem (University of Bristol, Bristol, UK). These human podocytes were maintained in the RPMI 1640 medium (Gibco™) supplemented with 10% heat-inactivated fetal bovine serum (FBS), insulin-transferrin-selenium-pyruvate supplement (ITSP; Gibco™), and antibiotics. Fresh media was provided once every two days. To induce differentiation, podocytes were maintained at 37 °C without ITPS (non-permissive conditions) for at least 2 weeks, and 0.05% trypsin was used to detach cells from the culture dishes [27].

### 2.2. Podocyte Treatment with IL-4 and Visualization and Quantification of Intracytoplasmic Actin Filaments

To identify whether IL-4 at a dose of 10 ng/mL could induce changes in intracytoplasmic actin filaments in podocytes, which is the main hallmark of MCNS pathogenesis, cytoplasmic actin filaments were visualized by immunofluorescence microscopy. To quantitatively compare the differences in expression, the number of actin filaments was counted in cells from each group (IL-4-treated, IL4+DEX-treated, vehicle-treated controls, and non-treated controls). Podocytes treated with IL-4 were compared with vehicle-treated podocytes and human podocytes at 37 °C without any treatment for 6 h. The cells were fixed with 4% formaldehyde in PBS for 15 min at room temperature (RT). Cells were permeabilized using 0.3% Triton X-100 in PBS for 10 min at RT. After adding PBS and subjecting the cells to washing steps, the cells were stained with rhodamine-phalloidin (1:500, Invitrogen) by incubating for 30 min at RT. Thereafter, PBS was used to conduct the washing steps again, and the nuclei were stained with DAPI (1:5000, Sigma-Aldrich, St. Louis, MO, USA) and mounted. Actin filaments and nuclei were visualized using fluorescence microscopy. Images were acquired, and the number of actin filaments per cell was counted by the point-counting method using the Image J software (version 1.50i; National Institutes of Health, Bethesda, MD, USA).

### 2.3. Podocyte Treatment with IL-4 with or without Dexamethasone

Podocytes were divided into three groups. The first group was treated with IL-4, the second group was treated with IL-4 plus dexamethasone (DEX), and the third group was control (non-treated podocytes). The cells were then incubated for 6 h. Each experiment was performed in triplicate.

In the first group, IL-4 (Peprotech Inc., Rocky Hill, NJ, USA) was administered to human podocytes at a dose of 10 ng/mL in serum-free RPMI 1640 at 37 °C. As the dissolving vehicle for IL-4, 1% bovine serum albumin (BSA) was used following the manufacturer’s instructions. The dose of IL-4 used herein was 10 ng/mL, which was used in a previous animal model in which 10 ng/mL IL-4 induced podocyte effacement as detected under electron microscopy [17]. In the second group, IL-4 was treated in the same manner as the first group, and DEX (Sigma-Aldrich, Saint Louis, MO, USA) was simultaneously added and incubated together. DEX was dissolved in distilled water according to the manufacturer’s instructions. Detailed quantitative assessments were performed by Xing et al. using various concentrations of DEX in which DEX concentrations between 10^−7^ and 10^−5^ M equated to therapeutic concentrations in vivo. Therefore, 10^−6^ M DEX was used to treat podocytes in our study [28]. The third group, considered as a control, was incubated for 6 h in 1% BSA, the vehicle for IL-4, because IL-4 and cytokines are already sent as BSA as a routinely used carrier protein.

### 2.4. Whole Transcriptome Sequencing

RNA sequencing of the three groups of podocytes was performed, and the differentially expressed genes (DEGs) between the groups were analyzed.

Podocytes after treatment and incubation were suspended in RNAlater^®^ (Invitrogen, Carlsbad, CA, USA) and stored at −20 °C until further analysis to prevent RNA degradation. Total RNA was purified using the RNeasy Mini Kit (Qiagen, Hilden, Germany), and DNase digestion was performed to prevent genomic DNA contamination. RNA integrity and quantity of each sample were evaluated using the Agilent 2100 Bioanalyzer (Agilent Technologies, Santa Clara, CA, USA). Using mRNA captured from total RNA by using oligo-dT beads, a library was constructed using the NEXTFLEX^®^ Rapid RNA-Seq Kit (PerkinElmer, Inc., Waltham, MA, USA), whereas the small RNA-Seq library was prepared using the NEXTFLEX^®^ Small RNA-Seq Kit (PerkinElmer Inc., Waltham, MA, USA) following the manufacturer’s instructions. Paired-end sequencing was performed using Illumina Nexseq550 (Illumina, San Diego, CA, USA) with a 100-bp read length after the quality control process, and the quantity of the libraries was assessed using the Agilent 2100 Bioanalyzer.

### 2.5. Data Analysis and Statistical Modeling

#### 2.5.1. Principal Component Analysis

Principal component analysis (PCA) was used as a statistical procedure to simplify the complexity of high-dimensional data while maintaining trends and patterns. PCA was performed for each of the individual samples to determine the overall patterns of the responding genes based on the RNA-seq expression profiles.

#### 2.5.2. Identification of DEGs

The primary analysis of high-throughput sequencing data was performed using TopHat, htseq, and DEGseq2 (version 1.10.0) [29]. In our study, raw FASTQ files were mapped to the UCSC hg19 reference genome using the TopHat software (version 1.4.0) and Bowtie software (version 2.3.5) [30]. Once the sequence reads were successfully mapped to the reference genome, the htSeq program, which contains powerful options for analyzing high-throughput sequencing data, was used to count the reads mapped to each gene. Once the count of the reads for each gene was calculated, the tags with markedly low counts were filtered out to prevent the intervention of weakly expressed tags. Then, a normalization process was applied to account for the differences in the number of reads produced in different sequencing runs, as well as technical biases caused by the library preparation protocols, sequencing platforms, and nucleotide compositions. To identify the DEGs, the DESeq2 package was used. Using the negative bionomical assumption, the coefficient of variance for each gene was estimated. Pairwise comparisons were performed between the control group and the two treatment groups to identify significant DEGs (adjusted *p*-value < 0.1).

#### 2.5.3. Construction of Protein-Protein Interaction Networks

The STRING 9.1 network database is one of the largest databases of direct (i.e., physical) and indirect (i.e., functional) protein–protein interactions and contains data from various sources, including genomic context predictions, high-throughput experiments, co-expression analyses, and existing databases [31]. The database comprises data on 9.6 million proteins obtained from more than 2031 organisms. This database was used to identify protein-protein interactions covering the identified DEGs from different comparisons. As our gene sets were represented by gene symbols, the Ensembl protein identifiers in the original STRING database were converted into gene symbols using mapping information in EntrezID.

#### 2.5.4. Gene Ontology Enrichment Analysis

Gene ontology (GO) enrichment analysis was performed for all DEGs obtained from each comparison. The enriched GO terms were identified using hypergeometric tests between DEGs from each comparison and GO-annotated biological pathway genes with a *p*-value of 0.05.

#### 2.5.5. Selection of the Genes of Potential Relevance

To identify the critical genes involved in IL-4-induced podocyte changes, DEGs were compared with those reported in previous investigations and databases. PodNet [31], a literature-based, expert-curated database of mouse podocytes, was used. Although PodNet is based on data obtained from mouse podocyte analyses, it has been regarded as a valuable tool for conducting interactome analysis in podocytes and for interpretation of podocyte expression data [31].

## 3. Results

### 3.1. Visualization of Intracytoplasmic Actin Filaments in Human Podocytes

**Figure 1:**
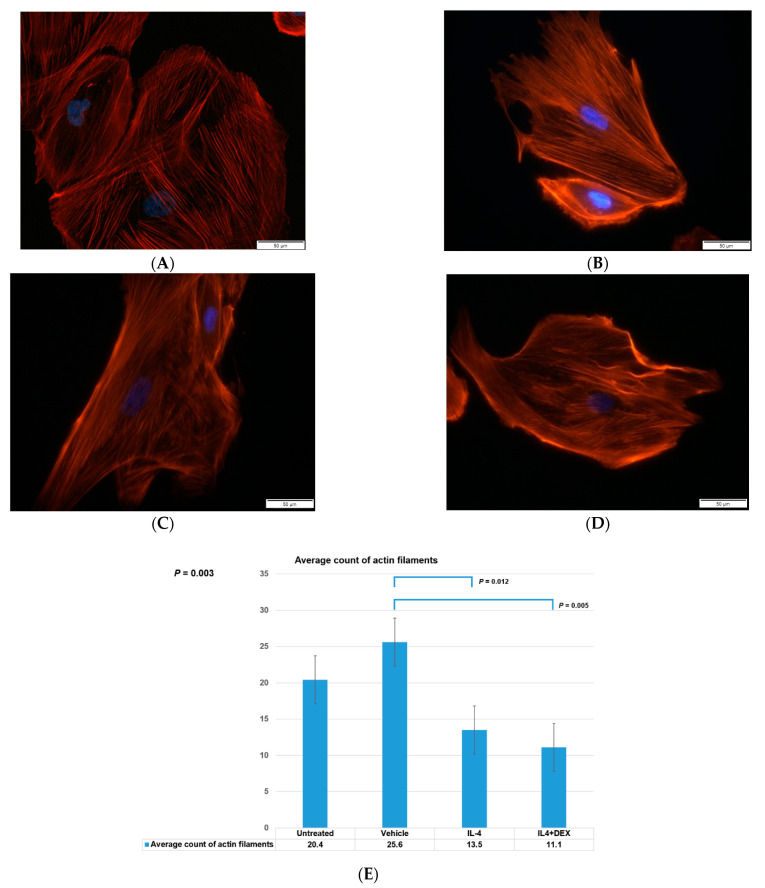
Intracytoplasmic actin filaments in podocytes. Rhodamine-Phalloidin stain for six hours revealed intracytoplasmic actin filaments (red) in (**A**) cultured human podocytes without any treatment, (**B**) vehicle-treated control podocytes, (**C**) IL-4-treated podocytes, and (**D**) IL-4+dexamethasone-treated cells. (**E**) shows the average number of actin filaments counted from each group. Statistically significant results are shown for *p* value. Blue marks indicate the nuclei. Abbreviations: IL-4, interleukin-4; DEX, dexamethasone.

We investigated the effect of IL-4 on intracytoplasmic actin filaments by immunofluorescence microscopy. The four groups of human podocytes, namely IL-4-treated cells, IL-4+DEX-treated cells, vehicle-treated controls, and non-treated controls, were compared. The IL-4-treated cells showed a decreased amount of intracytoplasmic actin filaments compared to the vehicle-treated and non-treated controls (Figure 1A–E).

### 3.2. PCA and Analysis of DEGs

**Figure 2:**
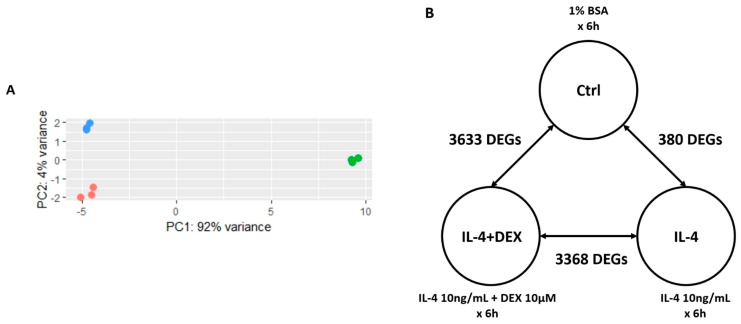
Principal component analysis (PCA) and differentially expressed genes (DEGs) between the podocyte groups. (**A**) PCA of human podocytes whole-transcriptome treated with interleukin-4 (red), interleukin-4 plus dexamethasone (green), and control (blue), and (**B**) the DEGs. Abbreviations: Ctrl, vehicle-treated control; IL-4, interleukin-4; DEX, dexamethasone.

Based on the data obtained from the above-mentioned experiment, which indicated that IL-4 expression induced changes in the major structural proteins of human podocytes, we performed transcriptome analysis. We studied podocytes in three groups, namely IL-4-treated cells, vehicle-treated controls, and podocytes co-treated with IL-4 and DEX. The results of PCA showed that IL-4-treated podocytes were highly distinct from the other groups, and over 90% of their variance was explained by PC1 (Figure 2A). The assessment of DEGs among the three groups of podocytes revealed the involvement of 3368 DEGs between IL-4-treated and IL-4+DEX-treated podocytes and 3633 DEGs between IL-4+DEX-treated and control groups (adjusted *p*-value < 0.1). Between IL-4-treated podocytes and controls, 380 DEGs were identified (Figure 2B). A total of 176 genes varied at the intersection of the three groups.

### 3.3. GO Pathways Related to the DEGs

**Figure 3:**
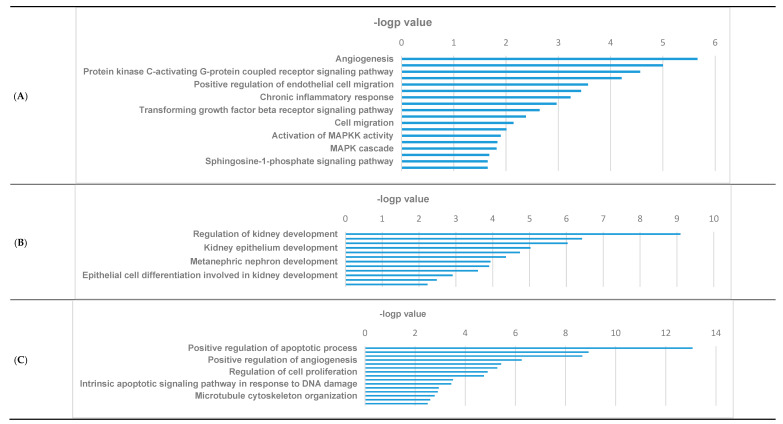

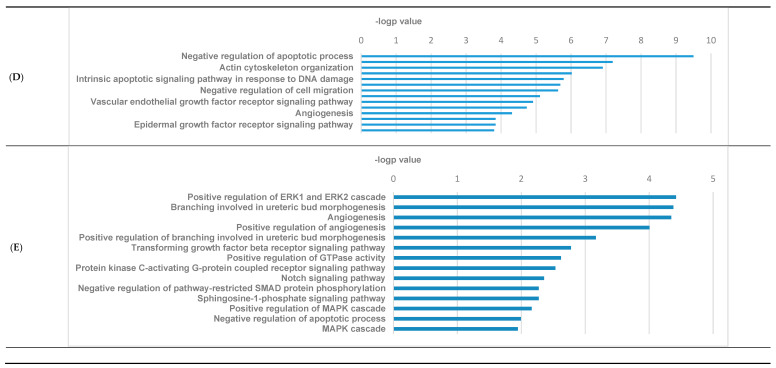
Gene ontology (GO) enriched pathyway analyses. (**A**) positively regulated DEGs between IL-4-treated podocytes and controls, (**B**) negatively regulated DEGs between IL-4-treated podocytes and controls, (**C**) most differentially expressed DEGs between IL-4-treated and IL-4+DEX-treated podocytes, (**D**) most differentially expressed DEGs between controls and IL-4+DEX-treated podocytes, and (**E**) most differentially expressed DEGs among the 176 overlapping DEGs in the three groups. Abbreviations: DEG, differentially expressed genes; IL-4, interleukin-4; DEX, dexamethasone.

To investigate the clinical significance of the DEGs among the three groups of podocytes, we performed GO enrichment analysis. Among the 380 DEGs between IL-4-treated and control groups, positively regulated pathways were related to angiogenesis, cell signaling, regulation, and migration (Figure 3A), while negatively regulated pathways were involved in renal development and morphogenesis (Figure 3B). Figure 3C illustrates the pathways for DEGs observed between IL-4-treated and IL-4+DEX-treated podocytes, thereby indicating the effects of DEX addition. Here, the pathways most differentially affected by the addition of DEX were those involved in the regulation of apoptosis, actin cytoskeleton organization, and cell regulation (Figure 3C). Likewise, between the control podocytes and those co-treated with IL-4 plus DEX, the considerably affected pathways comprised those involved in the regulation of apoptosis, cytoskeleton organization, and cell regulation (Figure 3D). Further, the pathways that were commonly different in the three comparison groups were related to morphogenesis, angiogenesis, and several signaling pathways involving extracellular signal-regulated kinase (ERK), Notch, and mitogen-activated protein kinase (MAPK) (Figure 3E).

The heatmap of genes that showed the highest variance among the three groups (IL-4/IL-4+DEX/control) is presented in Supplemental Figure S1. The top ranked positively or negatively regulated DEGs observed between IL-4-treated and vehicle-treated podocytes, and between IL-4-treated and IL-4+DEX-treated podocytes are summarized in Supplemental Figures S2 and S3. Although not all DEGs have been clearly elucidated for their roles exhibited in podocytes, the expression of a few genes has been previously reported to be associated with podocytes (Table 1).

**Figure 4:**
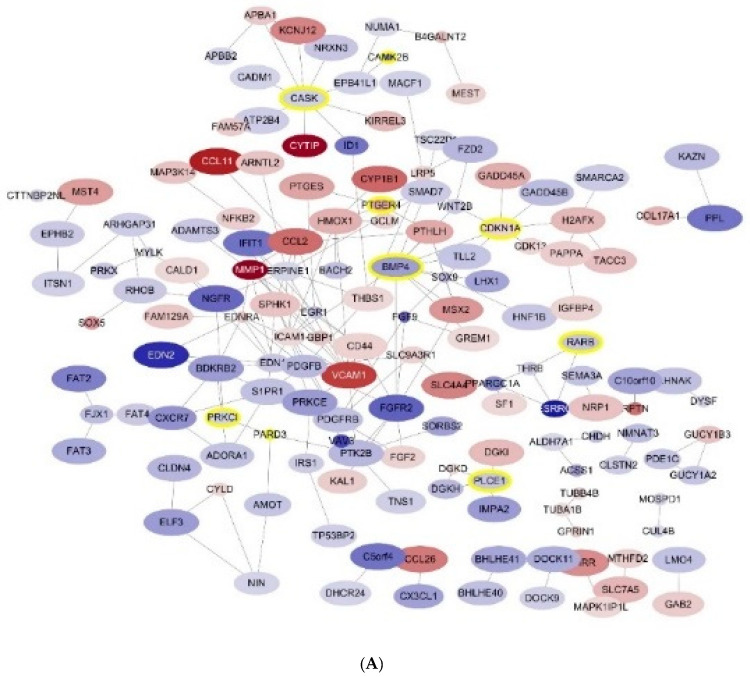

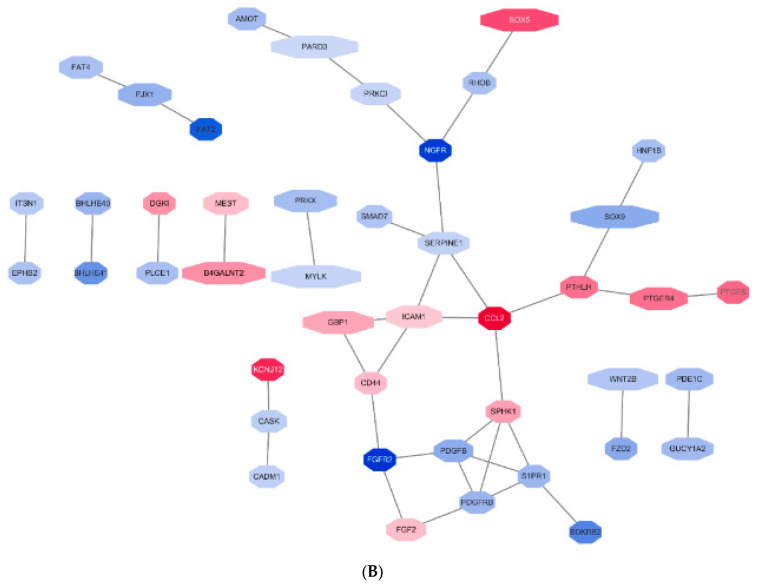
Protein-protein interaction (PPI) netwotk of differentially expressed genes (DEGs) (**A**) 380 DEGs between IL-4-treated podocytes and controls, and (**B**) 176 common DEGs among the three podocyte groups. Abbreviations: DEG, differentially expressed genes; IL-4, interleukin -4. positively regulated.

We investigated DEGs in the protein-protein interaction (PPI) network. Figure 4 shows the interaction of the 380 DEGs between IL-4-treated podocytes and controls (Figure 4A) and of 176 common DEGs in the three groups (Figure 4B).

### 3.4. Selection of the Genes of Potential Relevance to IL-4-Induced Pathogenesis of NS

**Figure 5:**
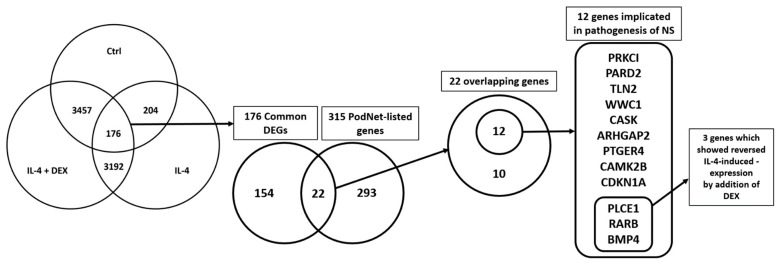
Overall flow of sorting out the possibly relevant genes related to IL-4-induced pathogenesis of nephrotic syndrome. Abbreviations: Ctrl, control; IL-4, interkleukin-4; DEX, dexamethasone; DEGs, differentially expressed genes; NS, nephrotic syndrome, PodNet, a protein-protein interaction network in mouse podocyte, published in 2013 [31].

To identify the critical genes involved in IL-4-induced podocyte changes, we compared the identified DEGs with those reported in previous studies and those existing in databases. We first compared the 176 commonly varied DEGs with 315 listed genes in PodNet [31]. We found 22 overlapping genes (7 upregulated and 15 downregulated expression levels; Supplemental Table S1). The gene expression patterns and the protein-protein interaction network of these 22 genes are presented in Supplemental Figure S4A–C). Second, for the overlapping 22 genes, we performed a literature search to explore the functional relevance reported in previous podocyte models. The overall selection process is summarized in Figure 5.

**Figure 6:**
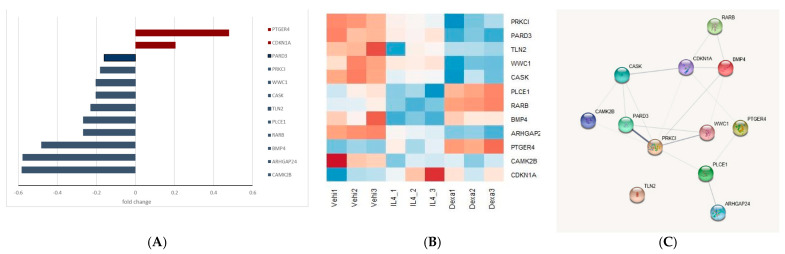
Features of the twelve common differentrially expressed genes (DEGs). (**A**) Expression profiles (red, positively regulated; green, negatively regulated), (**B**) Heat map, and (**C**) protein-protein interaction network of the 12 common DEGs.

Twelve out of the twenty-two DEGs were underpinned by previous studies using podocyte injury models. These DEGs included PRKCI, PARD2, TLN2, WWC1, CASK, ARHGAP2, PTGER4, CAMK2B, CDKN1A, PLCE1, RARB, and BMP4 (Figure 6A). Among them, the expression levels of PTGER4 and CDKN1A were upregulated, while those of the remaining were downregulated. The heatmap of the 12 genes graphically illustrates their expression (Figure 6B), and the PPI network for these 12 DEGs depicts their interaction (Figure 6C). All 12 genes showed positive or negative expression induced by IL-4 treatment (Supplementary Figure S5A–L).

**Figure 7:**
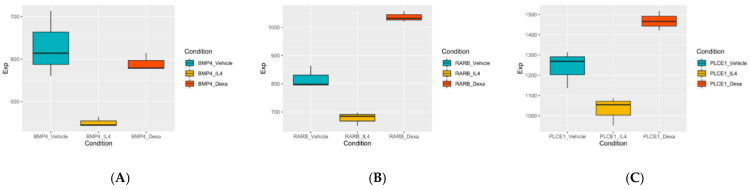
Gene expression of three genes which showed reversed expression when treated with dexamethasone (**A**) BMP4, (**B**) CAMK2B, and (**C**) PLCE1 (cyan: vehicle-treated control; yellow: IL-4 treated; red:IL-4+DEX-treated). Abbreviations: DEG, differentially expressed genes; IL-4, interleukin -4; DEX, dexamethasone.

Thereafter, we compared the expression of the 12 DEGs in the three podocyte groups and investigated the genes that showed IL-4-induced effects reversed by co-treatment with DEX. This process helped us to obtain data on three genes, namely BMP4, RARB, and PLCE1 (Figure 7). These genes were either positive in the negative direction of expression when exposed to IL-4 or exhibited opposite directional expression when treated with simultaneous DEX and IL-4.

Overall, 12 genes—PRKCI, PARD2, TLN2, WWC1, CASK, ARHGAP2, PTGER4, CAMK2B, CDKN1A, PLCE1, RARB, and BMP4—were differentially expressed when treated with IL-4. These genes were shown to be related to podocytopathy in previous studies using human podocyte models and were also listed in PodNet. Moreover, three of them seemed to exhibit reversed expression when co-treated with DEX.

**Figure 8:**
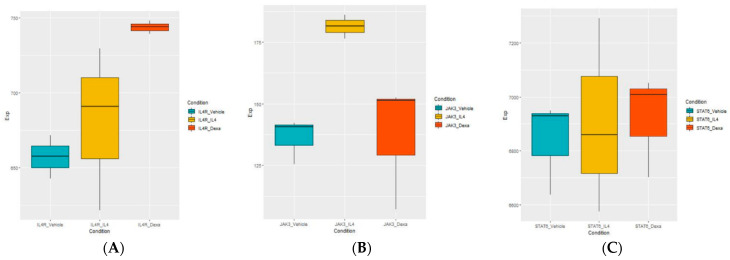
Gene expression of three genes which were involved in the signaling process of interleukin-4 (IL-4) (**A**) IL-4Rα, (**B**) JAK3, and (**C**) STAT6 (cyan: vehicle-treated control; yellow: IL-4 treated; red: IL-4+DEX-treated). Abbreviations: DEG, differentially expressed genes; IL-4, interleukin-4; DEX, dexamethasone; IL-4Rα, IL-4 receptor α subunit.

Additionally, to elucidate whether the subsequent signaling pathways were activated after IL-4 administration, the expression levels of IL-4 receptor α subunit (IL-4Rα), Janus kinase 3 (JAK3), and the signal transducer and activator of transcription 6 (STAT6) were further analyzed in the three groups (IL-4-treated, control, and IL-4- plus DEX-co-treated groups). There was no difference in the IL-4a gene expression among the three groups, but JAK3 was significantly upregulated (activated) in the IL-4-treated group compared to the control group, and this upregulation was suppressed by DEX addition. However, there was no difference in the STAT6 gene expression (Figure 8A–C).

## 4. Discussion

In this study, we analyzed the transcriptome of human podocytes and explored DEGs between the three groups treated with IL-4, IL-4 plus DEX, and the control group, respectively. Further, we performed GO analysis to investigate the pathways related to the DEGs. Among the DEGs, we investigated those that were supported by a literature-based database and experimental results reported by studies conducted using human podocyte models. We also examined whether the IL-4-induced expression levels of these genes were reversed when the podocytes were co-treated with DEX. To our knowledge, this is the first study to examine the effects of IL-4 treatment on the transcriptome of human podocytes.

We identified various biological pathways activated in podocytes after IL-4 treatment. A few notable cell-signaling pathways, including extracellular signal-regulated kinase (ERK), a member of the family of mitogen-activated protein kinases along with c-Jun N-terminal kinase, were identified. The ERK pathway has been implicated in podocyte injury in the progression of various glomerulopathies [32]. One study examined the phosphorylation of ERK in rat puromycin aminonucleoside nephropathy (PAN) model and mouse podocytes in vitro and concluded that the sustained activation of ERK was a crucial process in infliction of podocyte injury [33]. In our study, genes regulating the ERK pathway were positively expressed in the IL-4-treated podocytes compared to controls, supporting the results of a previous study [32], which suggested that ERK activation might be an important mechanism resulting in IL-4-induced podocyte damage. Similarly, TGF-β1 signaling was also shown to be increased in podocytes from PAN rats, suggesting that TGF-β1 might be involved in the pathogenic mechanisms of podocytopathies [33]. Our results were in line with those reported by a previous study, which stated that TGF-ß 1 signaling increased in IL-4-treated cells compared to controls. Notably, the expression levels of these two pathways were upregulated compared to controls in both IL-4-treated groups with or without DEX addition, indicating that DEX might not have inhibited these changes. The common pathways related to DEGs between the three groups included the Notch signaling pathway, which is related to cytoskeleton organization. The activation of the Notch pathway in podocytes has been suggested to be involved in glomerular injury. Niranjan et al. found that expression levels of genes belonging to the Notch pathway were regulated in patients and animal models of renal disease [34], showing that mice with conditional expression of active Notch 1 protein presented with massive albuminuria, glomerulosclerosis, and renal failure [34]. In support of this, the IL-4-treated group, compared to controls, showed negatively expressed DEGs in the pathways associated with renal development and morphogenesis, such as nephron and tubule morphogenesis and cytoskeleton organization. Our results imply that IL-4 may induce podocytopathy involving the activation of the Notch signaling pathway, which might downregulate the functions associated with cytoskeleton structures. Our experimental study on intracytoplasmic actin filaments in podocytes treated with IL-4 also supports the results obtained from GO pathway analysis, in that IL-4 treatment may affect the levels of major proteins that constitute the cytoskeleton.

**Figure 9:**
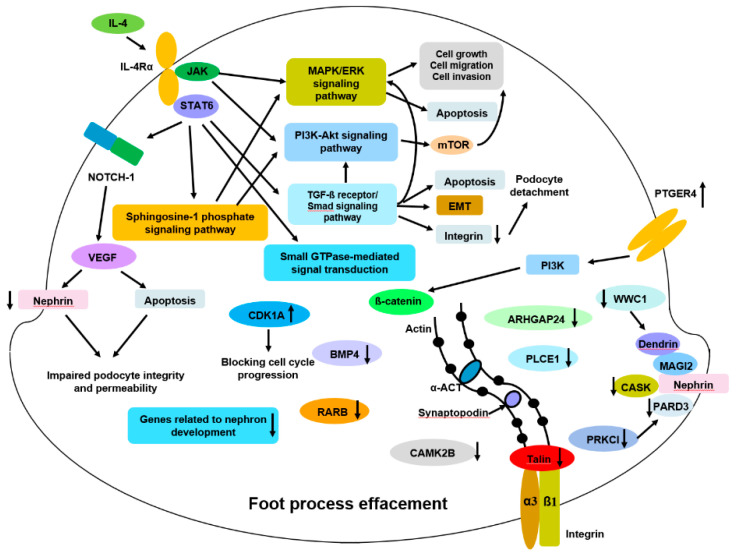
Proposed mechanisms of IL4-induced podocyte injury. Abbreviations: α-ACT, alpha actinin; ARHGAP24, Rho GTPase-activating protein 24; Akt, Protein kinase B; BMP4, Bone Morphogenetic Protein 4; CAMK2B, Calcium/Calmodulin Dependent Protein Kinase II Beta; CASK, peripheral plasma membrane protein; CDK1A, cyclin-dependent kinase inhibitor 1A; EMT, epithelial-mesenchymal-transition; ERK, extracellular signal-regulated kinases; GTP, guanosine triphosphate; IL-4, interleukin-4; IL-4Rα, IL-4 receptor alpha; JAK, Janus kinase; MAGI2, membrane associated guanylate kinase inverted-2; MAPK, mitogen-activated protein kinase; mTOR, mammalian target of rapamycin; PARD3, partitioning defective 3; PI3k, phosphoinositide 3-kinase; PLCE1, phospholipase C epsilon-1; PTGER4, prostaglandin E receptor 4; RARB, retinoic acid receptor beta; STAT6, signal transducer and activator of transcription 6; TGF-β, transforming growth factor beta; VEGF, vascular endothelial growth factor; WWC1, WW domain-containing protein 1 (a.k.a. kidney and brain scaffolding protein (KIBRA).

By comparing the common DEGs with genes listed in the PodNet database and those previously reported in relation to podocytopathy, we finally selected 12 genes, namely *PTGER4*, *CDKN1A*, *PARD3*, *PRKCI*, *WWC1*, *CASK*, *TLN2*, *PLCE1*, *RARB*, *BMP4*, *ARHGAP2*, and *CAMK2B*. The expression levels of the first two genes, *PTGER4* and *CDKN1A*, were upregulated, and those of the remaining were downregulated. Figure 9 summarizes the proposed mechanism of IL-4-induced podocyte injury involving these genes and pathways implicated in GO analyses.

Of the two positively expressed DEGs, *PTGER4* (prostaglandin E receptor 4) encodes for prostaglandin (PG) E4, and PGE2 is known to induce cyclooxygenase (COX)-2 expression in podocytes via the PGE4 receptor [35,36]. Enhanced expression of E-prostanoid receptor 4 (EP4) in cultured podocytes was shown to negatively affect podocyte adaptation to mechanical stretch [37], while the inhibition of EP4 attenuated the development of nephropathy in rodent models [38,39]. Additionally, EP4 inhibition prevented the TGF-ß1-induced dedifferentiation of glomerular podocytes [39]. In our study, the marked upregulation of *PTGER4* expression suggests that IL-4 may have triggered the EP4-induced pathogenic process. *CDKN1A* encodes cyclin-dependent kinase inhibitor 1A. In the kidney, the expression of cyclin I and p35 is specifically restricted to podocytes, and they limit podocyte apoptosis by activating cyclin-dependent kinase (Cdk)-5 [40,41]. Cyclin I functions as a cell survival factor by limiting apoptotic cell death in podocytes following injury [41]. Taniguchi et al. investigated their roles in a double cyclin I-p35 null murine model and demonstrated that the mutants showed markedly increased podocyte apoptosis when exposed to stress, which resulted in podocyte loss, proteinuria, and glomerulosclerosis [40]. They suggested that the activators of Cdk5, p35, and cyclin I might play a critical role in maintaining podocyte survival during stress. In support of this, our results showed the upregulation of *CDKN1A* expression, suggesting that increased cyclin I expression might be considered as a response to IL-4-induced stress injury.

We also observed that expression levels of 10 DEGs were downregulated. Partitioning defective 3 (*PARD3*) encodes polarity protein partitioning defective-3 protein, and polarity signaling through the atypical protein kinase C (aPKC), a par-polarity complex, is essential for the development and maintenance of podocyte architecture [42]. PARD3 has been suggested to play a functional role in the slit diaphragm [42], and its defect causes podocyte detachment [43]. In our study, *PARD3* expression was downregulated by IL-4 treatment, which suggested that IL-4 might cause a loss of polarity in podocytes. Along with PARD3, the aPKC complex is an essential regulator of podocyte morphology [43]. Protein kinase C Iota (PRKCI), which encodes protein kinase C iota, is an aPKC enzyme that contributes to cell proliferation [44]. As mentioned, structural maintenance of the slit diaphragm seems to be tightly regulated by polarity signaling pathways, and aPKC is an essential regulator of podocyte morphology [45]. It has been shown that aPKC interacts with the slit diaphragm complex and plays a critical role in the maintenance of the slit diaphragm and podocyte foot process [46]. The downregulation of *PRKCI* expression along with *PARD3* expression in our study suggests that IL-4 may induce dysregulation of critical polarity regulators. WW domain-containing protein 1 (WWC1), also known as kidney and brain scaffolding protein (KIBRA), is predominantly expressed in the kidney [47]. The knockdown of *WWC1* in an in vitro model promoted podocyte apoptosis by inducing nuclear relocation of dendrin protein [48] and impaired directed cell migration [49]. Conversely, KIBRA has also been shown to promote podocyte injury by inhibiting YAP signaling, by disrupting actin cytoskeletal dynamics [50], and by interacting with synaptopodin [51]. While the published results on the role of WWC1 in podocytes appear contradictory, *WWC1* expression was downregulated in the present study. *CASK* is a calcium/calmodulin-dependent serine protein kinase (CASK), a scaffolding protein that participates in the maintenance of polarized epithelial cell architecture by linking membrane proteins and signaling molecules to the actin cytoskeleton [52]. CASK has been shown to induce proteinuria and podocyte foot process effacement in a mouse model [53]. Circulating CASK has also been associated with post-transplantation recurrence of focal segmental glomerulosclerosis (FSGS) [53]. It may be interpreted that the downregulation of *CASK* expression by IL-4 treatment results in the impaired maintenance of epithelial structures. *TLN2* is one of the two talin genes that vertebrates possess, encoding talin-2 [54]. Talin-1 and talin-2 are cytoplasmic adapter proteins that are essential for integrin-mediated cell adhesion to the extracellular matrix and signaling via interaction with kidney ankyrin repeat-containing protein (KANK) [54]. Talins have been proposed to activate integrins and link them to the actin cytoskeleton. Mice lacking talins in the developing ureteric bud developed kidney agenesis and collecting duct cells of these animals exhibited severe cytoskeletal adhesion, and polarity defects [55]. We speculate that the downregulation of *TLN2* expression by IL-4 treatment in this study supports the previous observations and our experiment, which highlights a decreased number of actin filaments after IL-4 treatment. *ARHGAP24* is a kidney structure-related gene encoding Rho-GAP 24, and its expression is upregulated during podocyte differentiation [56]. Knockdown of *ARHGAP24* results in increased levels of active Rac1 and Cdc42, which consequently induces NS [56]. It could be suggested that IL-4 treatment decreased the expression of *ARHGAP24* in this study and caused podocyte injury. The gene *CAMK2B* encodes calcium/calmodulin-dependent protein kinase 2 (CaMK II), which participates in calcium signaling in podocyte injury [57]. CaMK II is involved in angiotensin (Ang) II-induced podocyte injury [58]. Specifically, Ang II can promote canonical Wnt upregulation, which is mediated by CaMK II/CREB signaling activation, and the inhibition of this signaling cascade ameliorated Ang II-induced podocyte injury in vitro and in vivo [58]. In this study, *CAMK2B* expression was most remarkably downregulated.

Notably, the IL-4-induced expression of three genes (*PLCE1*, *RARB*, and *BMP4*) was reversed when podocytes were co-treated with DEX. *PLCE1* encodes phospholipase C epsilon. The gene has been identified to cause monogenic NS, and affected individuals present with histologic characteristics of FSGS or diffuse mesangial sclerosis (DMS) [59,60]. PLCE1 interacts with IQ motif-containing GTPase-activating protein 1 (IQGAP1), which is known to directly interact and co-localize with nephrin in podocytes [59]. The absence of *PLCE1* expression halted glomerular development in a rodent model [59]. The downregulation of *PLCE1* expression reported in our study supports previous studies on exploration of podocyte changes, and IL-4 treatment may have induced this process. *RARB* encodes the retinoic acid receptor (RAR) beta, and nuclear receptors, including retinoic acid receptor (RAR), have been proposed to play a protective role in podocytes [61]. Using an RAR ß2 agonist improved podocyte effacement in a diabetic nephropathy model [62]. In our study, the downregulated expression of *RAR* during IL-4 treatment supported this finding. *BMP4* encodes bone morphogenetic protein 4, which exerts multiple biological effects on the morphogenesis of the kidney and ureters [63]. Podocyte-derived BMP reportedly plays an important role in glomerular capillary formation [63]. In our study, IL-4 decreased *BMP4* expression. The fact that *PLCE1* expression was reversed by steroid treatment is of clinical interest because *PLCE1* expression is known to cause monogenic NS, which is theoretically not immune-based [59]. Downregulated expression of *RARB*, which plays a protective role in podocytes, and *BMP4*, which is involved in kidney morpho- and angiogenesis, were also reversed by DEX treatment. Our findings suggest that these three DEGs could be implicated in the pathophysiology of NS in response to steroids.

Our study has several limitations. First, as our study was not a proof-of-concept study for targeted genes with a particular hypothesis but was rather a screening for the potential genes expressed in human podocytes affected by IL-4 treatment, the results of RNA sequencing were not confirmed by targeted qPCR and/or western blotting. This process of validation is warranted to elucidate the functional significance of the genes. The present study can only propose that these genes are possibly implicated in the IL-4-induced pathogenesis of NS. Second, IL-4 and DEX were not investigated for dose or time-dependence. We used a dose of 10 ng/mL for IL-4, which is known to cause NS in animal models, but higher doses of IL-4 might have led to the induction of pronounced changes in DEGs in podocytes. Likewise, the protective effect of DEX on IL-4-induced changes may have resulted in different outcomes at different doses. Additionally, the podocytes were uniformly incubated for 6 h after treatment in this study. Analyses at different time points would have yielded different results.

Nevertheless, this study demonstrated a negative effect of IL-4 exerted on human podocytes, which supported its potentially pathogenic role in NS, as suggested in the literature. First, immunohistochemistry revealed that incubation with IL-4 for 6 h led to a decrease in the number of actin filaments. Second, transcriptome analysis showed that IL-4 induced changes in gene expression. Third, genes that were supported by previous evidence were identified. The expression of three of these genes could be reversed by simultaneous steroid treatment. Since MCNS is usually responsive to steroid treatment, we suggest that these three genes may play a role in its pathogenesis. Further in vitro and in vivo studies are necessary to investigate the mechanisms by which the aberrant expression of these genes induces podocytopathy.

## 5. Conclusions

In this study, we propose few genes of interest that have possible implications in IL-4-induced pathogenesis of NS. RNA sequencing revealed that IL-4 induced changes in the expression of genes in human podocytes. GO analysis revealed certain pathways of relevance in NS, such as pathways related to podocyte cytoskeleton organization. Based on the DEG analysis, 12 genes were found to be involved in IL-4-induced changes in podocytes, and 3 genes, *BMP4*, *RARB*, and *PLCE1*, showed reversed expression when the podocytes were co-treated with IL-4 and DEX. The clinical significance of these genes remains to be explored; however, they might be involved in the induction of MCNS, as their expression levels were reversed by steroid treatment. This study suggests that molecular players may be involved in the induction of IL-4 mediated podocyte changes in an in vitro setting.

## Figures and Tables

**Table 1:** Genes associated with podocytes in the main Gene Ontology (GO) pathways.

Gene Ontology (GO) Pathway	Genes
Positive Regulation of ERK1 and ERK2 cascade	FGFR2, FGF2, PDGFB, ICAM1, GCNT2, CCL2, CD44, SEMA7A, PDGFRB, CX3CL1
Positive Regulation of MAPK cascade	FGFR2, PDGFB, C1QTNF1, WWC1, NGFR
Negative Regulation of apoptotic process	PDGFRB, PAK2, CD44, SPHK1, PRKCI, MALT1, NGFR, RARB, GREM1, APBB2, SOX9

Abbreviations: APBB2, amyloid beta A4 precursor protein-binding family B member 2; C1QTNF1, complement C1q tumor necrosis factor-related protein 1; CCL2, C-C Motif Chemokine Ligand 2; CD44, cluster of differentiation 44; CX3CL1, C-X3-C Motif Chemokine Ligand 1; FGF2, fibroblast growth factor 2; FGFR2, fibroblast growth factor receptor 2; GCNT2, glucosaminyl (*N*-acetyl) transferase 2; GREM1, Gremlin 1; ICAM1, Intercellular Adhesion Molecule 1; MALT1, mucosa-associated lymphoid tissue lymphoma translocation 1; NGFR, Nerve Growth Factor Receptor; PAK2, p21-activated kinase 2; PDGFB, platelet derived growth factor subunit B; PDGFRB, platelet derived growth factor receptor subunit B; PRKCI, Protein kinase C iota; RARB, retinoic acid receptor beta; SEMA7A, Semaphorin 7A; SOX9, SRY-Box Transcription Factor 9; SPHK1, Sphingosine kinase 1; WWC1, WW domain-containing protein 1.

## Data Availability

The data presented in this study are available on request from the corresponding author.

## Supplementary Materials

The following are available online at https://www.mdpi.com/2077-0383/10/3/496/s1, Table S1: 22 genes overlapping with 380 genes differentially identified between the IL-4- and vehicle-treated groups and with genes listed in PodNet; Figure S1: Heatmap of human podocytes whole-transcriptome sequencing of genes which showed top variance between the IL-4/IL-4+DEX treated and control groups of podocytes; Figure S2: Top-listed DEGs that were (A) negatively and (B) positively regulated in IL-4-treated-podocytes compared to vehicle-treated controls; Figure S3: Top-listed DEGs which were (A) negatively and (B) positively changed in IL-4 treated-podocytes compared to IL-4 plus dexamethasone treated podocytes; Figure S4: (A) Heatmap of 22 genes overlapping with 176 common differentially identified genes (DEGs) and genes listed in the PodNet, (B) positive- or negative expression of the 22 DEGs, (C) Protein-protein interaction network for the 22 DEGs; Figure S5: Gene expression levels of the following 12 DEGs among the three groups.
