# Supplementary material for: The Effect of Interleukin-4 and Dexamethasone on RNA-Seq-Based Transcriptomic Profiling of Human Podocytes: A Potential Role in Minimal Change Nephrotic Syndrome
Source: J Clin Med. 2021 Feb 1;10(3):496. doi: 10.3390/jcm10030496 (PMC7866993; doi:10.3390/jcm10030496)
Supplement: Supplementary file 1 [file jcm-10-00496-s001.pdf]

Supplementary Materials

Supplemental Table 1. 22 genes overlapping with 176 common differentially identified genes (DEGs) and genes listed in the PodNet.

Supplemental Figure 1. Heatmap of human podocytes whole-transcriptome sequencing of genes which showed top variance between the IL-4/IL-4+DEX treated and control groups of podocytes.

Supplemental Figure 2. Top-listed DEGs which were (A) negatively and (B) positively changed in IL-4 treated-podocytes compared to vehicle-treated controls.

Supplemental Figure 3. Top-listed DEGs which were (A) negatively and (B) positively changed in IL-4 treated-podocytes compared to IL-4 plus dexamethasone treated podocytes.

Supplemental Figure 4. (A) Heatmap of 22 genes overlapping with 176 common differentially identified genes (DEGs) and genes listed in the PodNet, (B) positive- or negative expression of the 22 DEGs, (C) Protein-protein interaction network for the 22 DEGs.

Supplemental Figure 5. Gene expression levels of 12 DEGs in three podocyte groups (A) BMP4, (B) CAMK2B, (C) CDKN1A, (D) PLCE1, (E) RARB, (F) PTGER4, (G) PARD3, (H) PRKCI, (I) WWC1, (J) CASK, (K) TLN2, (L) ARHGAP2.

Supplemental Table S1. 22 genes overlapping with 176 common differentially identified genes (DEGs) and genes listed in the PodNet

ID	Gene Name
ARHGAP24	Rho GTPase activating protein 24(ARHGAP24)
SH2D4A	SH2 domain containing 4A(SH2D4A)
SMAD7	SMAD family member 7(SMAD7)
WWC1	WW and C2 domain containing 1(WWC1)
ANGPTL2	angiopoietin like 2(ANGPTL2)
BMP4	bone morphogenetic protein 4(BMP4)
CAMK2B	calcium/calmodulin dependent protein kinase II beta(CAMK2B)
CASK	calcium/calmodulin dependent serine protein kinase(CASK)
CDKN1A	cyclin dependent kinase inhibitor 1A(CDKN1A)
KIRREL3	kin of IRRE like 3 (Drosophila)(KIRREL3)
NRP1	neuropilin 1(NRP1)
PAK2	p21 (RAC1) activated kinase 2(PAK2)
PARD3	par-3 family cell polarity regulator(PARD3)
PLCE1	phospholipase C epsilon 1(PLCE1)
PKD2	polycystin 2, transient receptor potential cation channel(PKD2)
PTGER4	prostaglandin E receptor 4(PTGER4)
PRKCE	protein kinase C epsilon(PRKCE)
PRKCI	protein kinase C iota(PRKCI)
RARB	retinoic acid receptor beta(RARB)
SEMA3A	semaphorin 3A(SEMA3A)
SYNPO	synaptopodin(SYNPO)
TLN2	talin 2(TLN2)

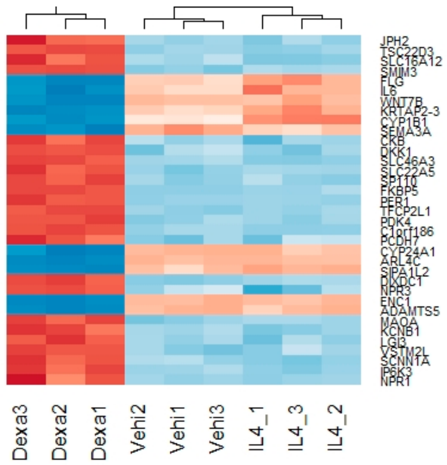

Supplemental Figure S1. Heatmap of human podocytes whole-transcriptome sequencing of genes which showed top variance between the IL-4/IL-4+DEX treated and control groups of podocytes

(A) **(B)**

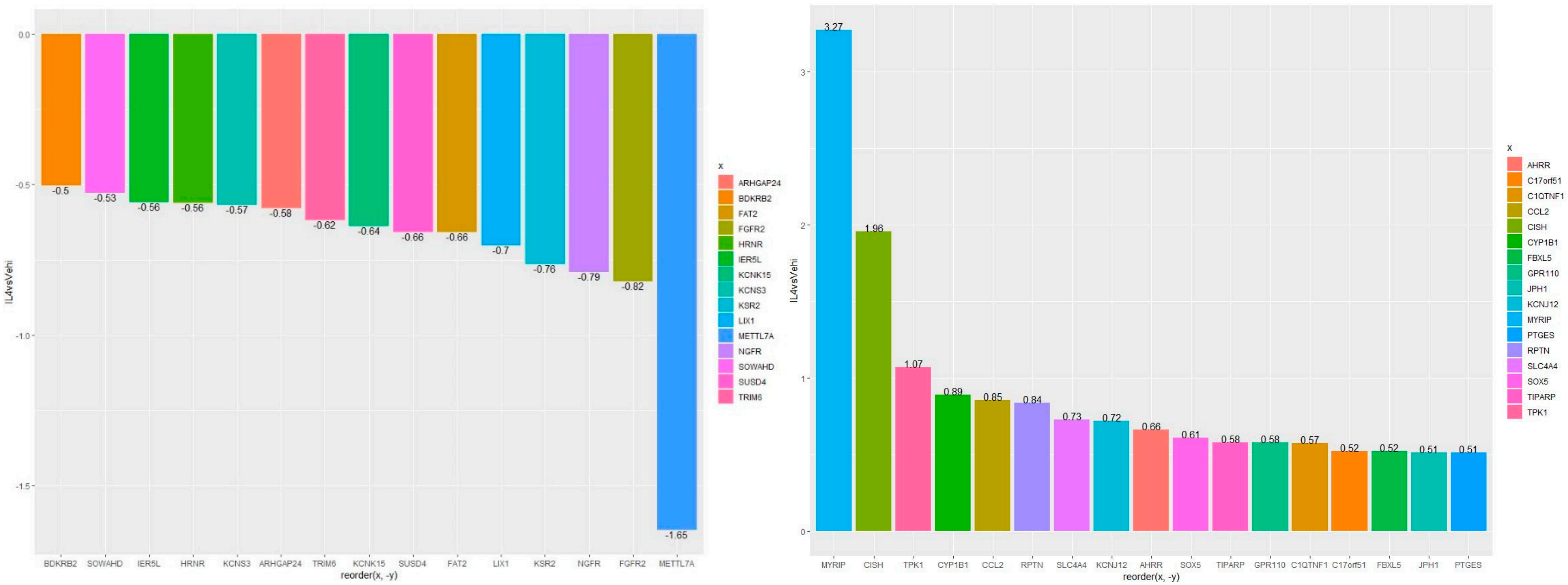

Supplemental Figure S2. Top-listed DEGs which were (A) negatively and (B) positively changed in IL-4 treated-podocytes compared to vehicle-treated controls.

(A) (B)

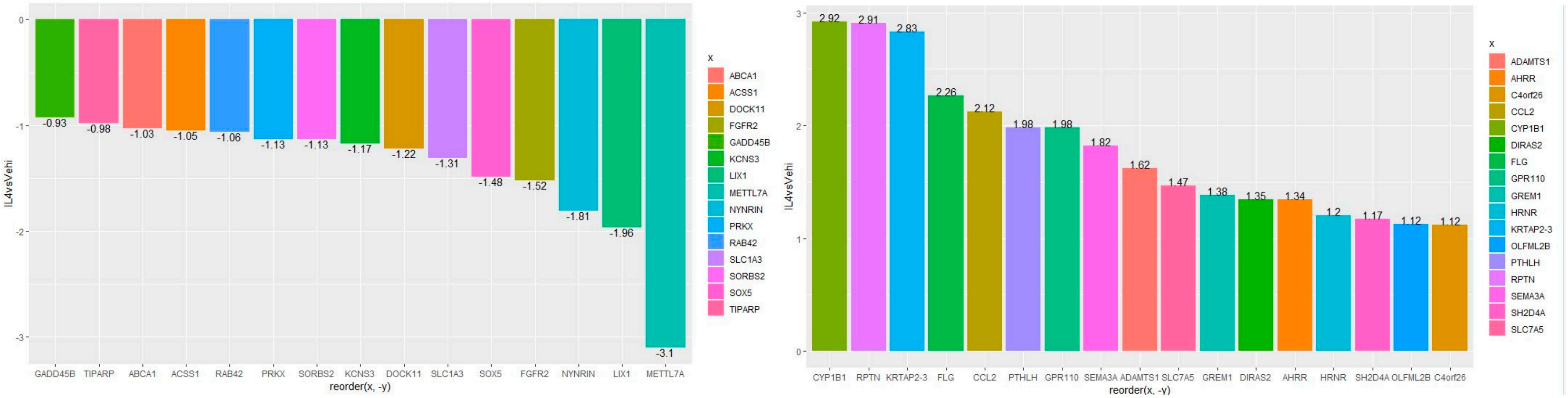

Supplemental Figure S3. Top-listed DEGs which were (A) negatively and (B) positively changed in IL-4 treated-podocytes compared to IL-4 plus dexamethasone treated podocytes.

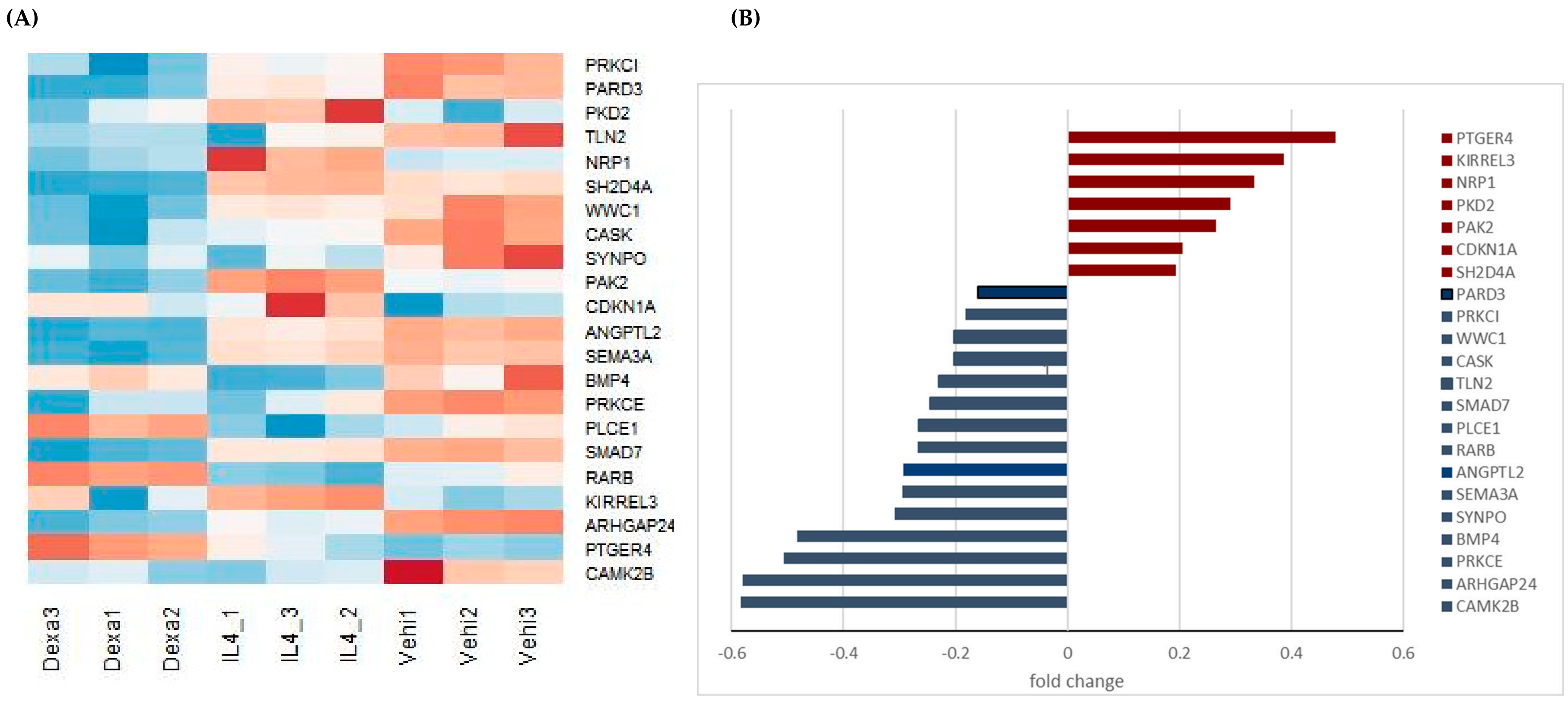

(A)
PRKCI
PARD3
PKD2
TLN2
NRP1
SH2D4A
WWC1
CASK
SYNPO
PAK2
CDKN1A
ANGPTL2
SEMA3A
BMP4
PRKCE
PLCE1
SMAD7
RARB
KIRREL3
ARHGAP24
PTGER4
CAMK2B
Dexa3
Dexa1
Dexa2
IL4_1
IL4_3
IL4_2
Vehi1
Vehi2
Vehi3
(B)
PTGER4
KIRREL3
NRP1
PKD2
PAK2
CDKN1A
SH2D4A
PARD3
PRKCI
WWC1
CASK
TLN2
SMAD7
PLCE1
RARB
ANGPTL2
SEMA3A
SYNPO
BMP4
PRKCE
ARHGAP24
CAMK2B
-0.6
-0.4
-0.2
0
0.2
0.4
0.6
fold change

(C)

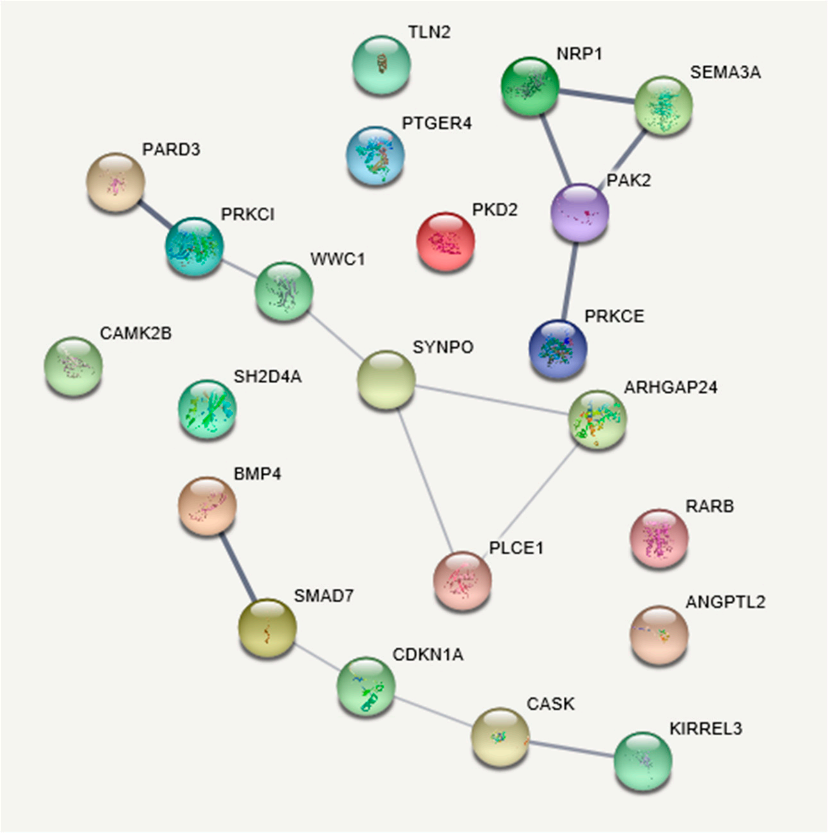

Supplemental Figure S4. (A) Heatmap of 22 genes overlapping with 176 common differentially identified genes (DEGs) and genes listed in the PodNet, (B) positive- or negative expression of the 22 DEGs, (C) Protein-protein interaction network for the 22 DEGs.

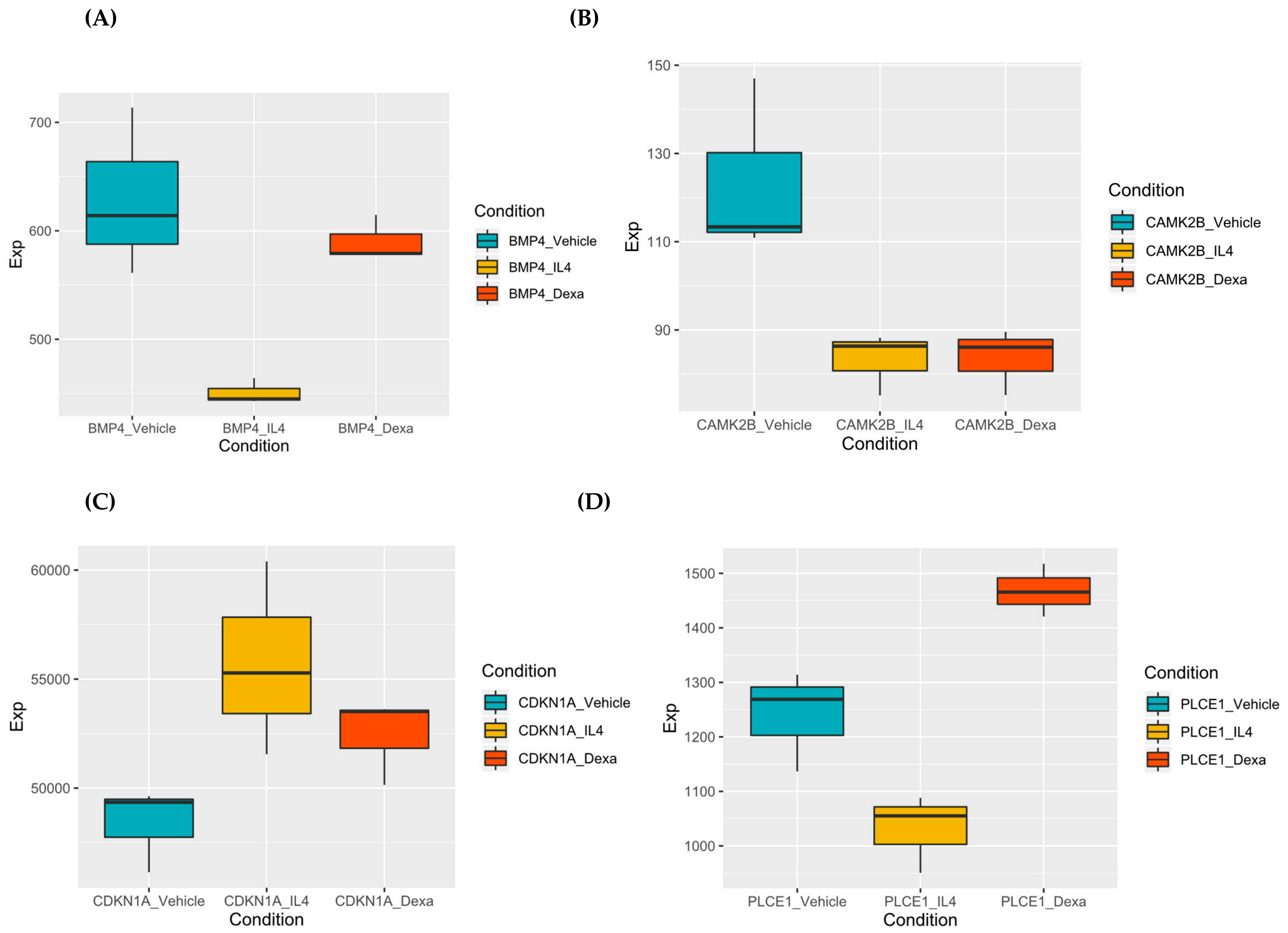
(A)
700
600
500
Exp
BMP4_Vehicle
BMP4_IL4
BMP4_Dexa
Condition
Condition
BMP4_Vehicle
BMP4_IL4
BMP4_Dexa
(B)
150
130
110
90
Exp
CAMK2B_Vehicle
CAMK2B_IL4
CAMK2B_Dexa
Condition
Condition
CAMK2B_Vehicle
CAMK2B_IL4
CAMK2B_Dexa
(C)
60000
55000
50000
Exp
CDKN1A_Vehicle
CDKN1A_IL4
CDKN1A_Dexa
Condition
Condition
CDKN1A_Vehicle
CDKN1A_IL4
CDKN1A_Dexa
(D)
1500
1400
1300
1200
1100
1000
Exp
PLCE1_Vehicle
PLCE1_IL4
PLCE1_Dexa
Condition
Condition
PLCE1_Vehicle
PLCE1_IL4
PLCE1_Dexa

(E)

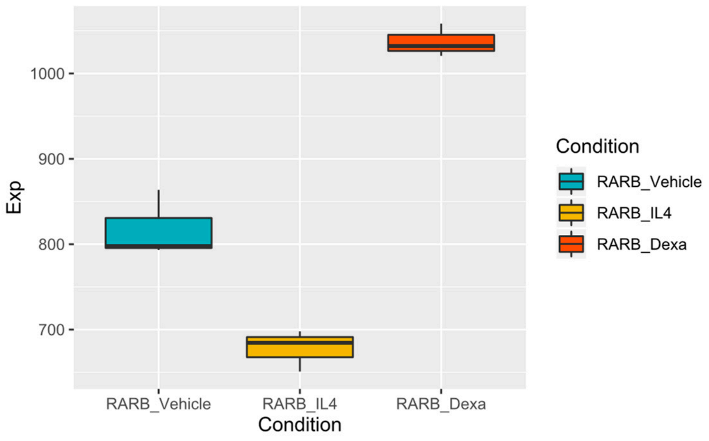
1000
900
800
700
Exp
RARB_Vehicle
RARB_IL4
RARB_Dexa
Condition
Condition
RARB_Vehicle
RARB_IL4
RARB_Dexa

(F)

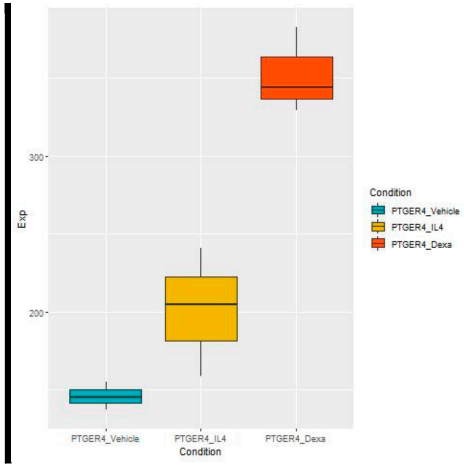
300
200
Exp
PTGER4_Vehicle
PTGER4_IL4
PTGER4_Dexa
Condition
Condition
PTGER4_Vehicle
PTGER4_IL4
PTGER4_Dexa

(G)

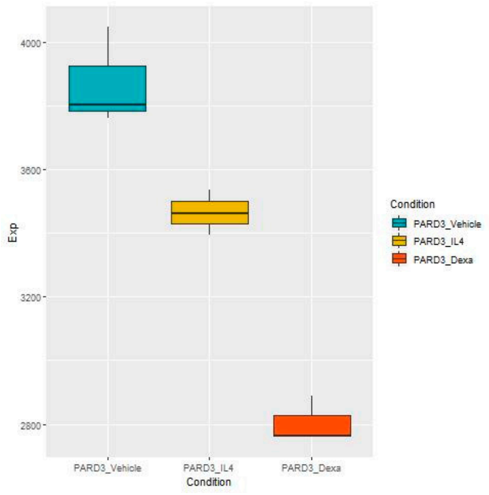
4000
3600
3200
2800
Exp
PARD3_Vehicle
PARD3_IL4
PARD3_Dexa
Condition
Condition
PARD3_Vehicle
PARD3_IL4
PARD3_Dexa

(H)

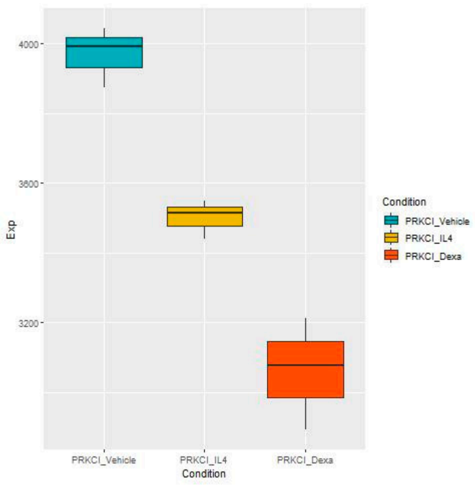
4000
3600
3200
Exp
PRKCI_Vehicle
PRKCI_IL4
PRKCI_Dexa
Condition
Condition
PRKCI_Vehicle
PRKCI_IL4
PRKCI_Dexa

(I)

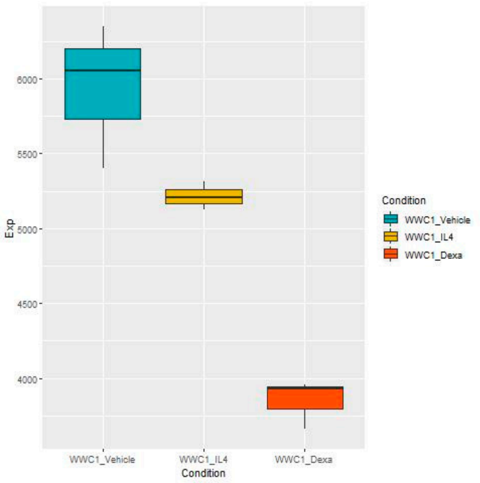

6000
5500
5000
4500
4000
Exp
WWC1_Vehicle
WWC1_IL4
WWC1_Dexa
Condition
Condition
WWC1_Vehicle
WWC1_IL4
WWC1_Dexa

(J)

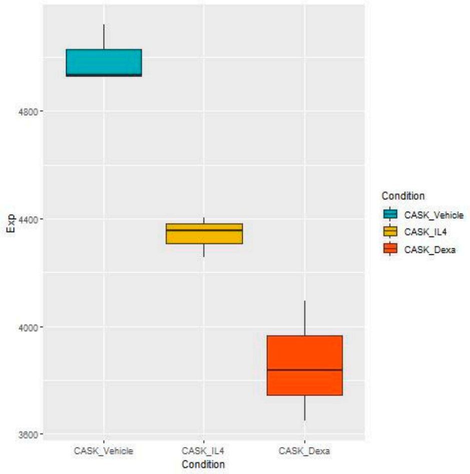

4800
4400
4000
3600
Exp
CASK_Vehicle
CASK_IL4
CASK_Dexa
Condition
Condition
CASK_Vehicle
CASK_IL4
CASK_Dexa

(K)

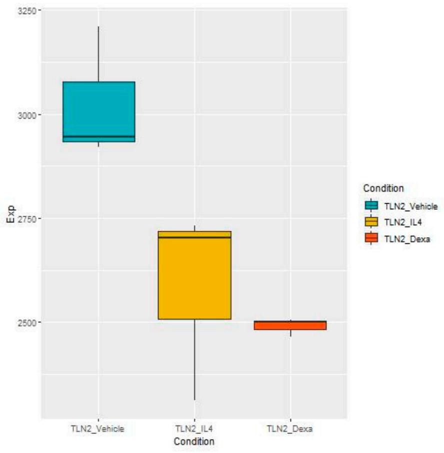

3250
3000
2750
2500
Exp
TLN2_Vehicle
TLN2_IL4
TLN2_Dexa
Condition
Condition
TLN2_Vehicle
TLN2_IL4
TLN2_Dexa

(L)

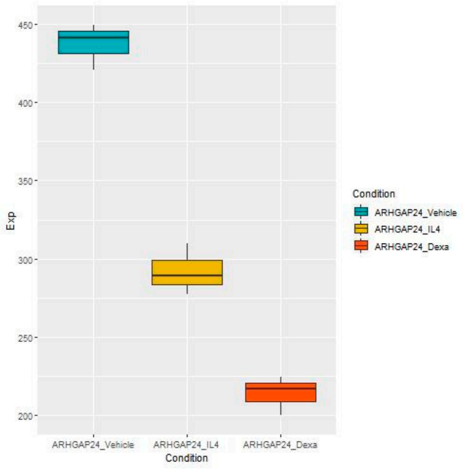

450
400
350
300
250
200
Exp
ARHGAP24_Vehicle
ARHGAP24_IL4
ARHGAP24_Dexa
Condition
Condition
ARHGAP24_Vehicle
ARHGAP24_IL4
ARHGAP24_Dexa

Supplemental Figure S5. Gene expression levels of 12 DEGs in three podocyte groups. (A) BMP4, (B) CAMK2B, (C) CDKN1A, (D) PLCE1, (E) RARB, (F) PTGER4, (G) PARD3, (H) PRKCI, (I) WWC1, (J) CASK, (K) TLN2, (L) ARHGAP2.
